# The use of anterior cervical interbody spacer with integrated fixation screws for management of cervical disc disease

**DOI:** 10.1051/sicotj/2019002

**Published:** 2019-02-08

**Authors:** Ehab Adel El Baz, Ahmed Maher Sultan, Ahmed Samir Barakat, Wael Koptan, Yasser ElMiligui, Hesham Shaker

**Affiliations:** 1 Agouza Armed Forces Spine Surgery Center Farid Shawky Street Giza Egypt; 2 Orthopedics and Traumatology Department, Faculty of Medicine, Cairo University Kasr Alainy Street Cairo Egypt

**Keywords:** Cervical disc disease, Integrated interbody spacer with zero-profile, Dysphagia after cervical fusion, ACDF, Cervical lordosis and cage subsidence

## Abstract

*Introduction*: Integrated cage and screw designs were introduced for anterior cervical discectomy and fusion (ACDF) and allegedly are superior to anterior plating due to their minimal anterior profile.

*Methods*: A descriptive study was designed as a prospective case series of 25 patients (30 operated discs) with cervical disc disease treated with a zero-profile cage, and followed up for an average of 16 months (range 12 –18 months). Functional assessment was done with the Neck Disability Index (NDI) and Visual analog scale (VAS) scores for arm and neck pain. Furthermore, Nurick’s classification system for myelopathy based on gait abnormalities was documented. Radiological fusion was confirmed with plain X-rays and when indicated with a CT scan at 12 months postoperatively. Dysphagia was classified according to the Bazaz criteria.

*Results*: VAS for neck and arm pain, NDI, and Nurick Score immediately improved postoperatively and remained so at 12-month follow-up. Fusion was achieved in 19 patients (95%) at six months and in 20 patients (100%) of the single-level group at one year. At six months 80% (four patients) and at 12 months 100% (five patients) showed complete union in the double-level group.

No evidence of cage subsidence was noted radiographically.

*Discussion*: The favorable lordosis and fusion rates of the low-profile integrated device are consistent with ACDF using anterior plating. Additionally, improved pain and an acceptable rate of dysphagia support the use of integrated interbody spacers for use in ACDF procedures.

## Introduction

In degenerative conditions of the cervical degenerative disc, anterior cervical decompression and fusion surgery or cervical disc replacement are indicated when nonoperative therapies fail [1].

Anterior cervical discectomy and fusion (ACDF) is considered the operative “gold standard” for patients, especially in whom a cervical disc prosthesis is contraindicated [2].

The mechanical goal of ACDF is to eliminate motion between adjacent vertebrae by forming a solid bony union which is obtained by minimizing intervertebral motion during the fusion phase. Furthermore, the position of any interbody graft or spacer should be maintained to prevent its extrusion, irritation of surrounding tissues, and to allow union with the adjacent vertebrae [3].

Still, some surgeons prefer to add an anterior plate in fusion procedures to increase stability and reduce cervical kyphosis, thereby intending to increase fusion and reduce failure rates, particularly in multilevel procedures [3].

Nevertheless, the addition of a plate is not without side effects, despite the profile of current anterior plates being thinner than that of earlier designs. In the early postoperative period, 2%–67% of the patients may complain of dysphagia [4]. During the first three months after surgery most of these symptoms disappear spontaneously, however complete recovery does not occur in all patients [4] and not all patients recover completely from swallowing problems. The incidence of chronic dysphagia-related symptoms after ACDF ranges from 3% to 21% [5]. Yet, the underlying pathological mechanism of postoperative dysphagia is unknown, but it has been associated with the ventral prominence associated with plate and screw constructs, the intraoperative esophageal retraction, subsequent adhesions, and instrumentation of the cervical spine [6].

Park et al. [7] demonstrated a higher incidence of adjacent-level degenerations if an additional plate was used stating that this was consistent with the inappropriately sized or misaligned plates interfering with the adjacent-level disc space. Yang et al. [8] supported this by demonstrating lower rates of adjacent-level degeneration when performing ACDF without plates.

The major limitations of constrained plates are their stiffness and intrinsic stress-shielding resulting in less frequent incorporation of the graft and a greater incidence of pseudarthrosis, which led to the development of load-sharing (dynamic) cervical plates [9].

Further development integrating contemporary biomechanical data resulted in a low-profile cervical implant system that combines a plate and a cage in one polyetheretherketone (PEEK) or titanium device for stand-alone anterior interbody fusion procedures [7].

A decade ago, low-profile anchored cages with integrated screw fixation appeared which secure the spacer directly to the endplates. The anchored spacer devices have been shown to provide biomechanical stability comparable to constructs that use an anterior plate to supplement an interbody cage in the presence of intact posterior soft tissue and bony structures [9,10].

Potentially these implants could decrease dysphagia, lower risk of migration, preserve or even restore cervical lordosis, and lower incidence of adjacent segment degeneration [10].

This study evaluates the feasibility, advantages, and drawbacks of cervical interbody spacer with integrated fixation screws in the management of anterior cervical fusion procedures.

## Methods

After obtaining institutional Ethics Committee approval, all patients signed an informed and detailed consent describing the procedure, alternative treatment methods, and possible complications.

This study was conducted between September 2013 and August 2016 at Agouza Armed Forces Spine Surgery Center, Giza, Egypt. Patients were subjected to a thorough preoperative clinical examination, which included demographic data, a detailed history of disease progression, neurological and radiological assessments including an antero-posterior, lateral X-rays, flexion, and extension views of the cervical spine, magnetic resonance imaging (MRI), and pre-operative Visual analog scale (VAS) and Neck Disability Index (NDI) scoring.

Patients were followed up postoperatively for a minimum period of one year both clinically and radiologically.

### Inclusion criteria

Inclusion criteria were symptomatic cervical disc disease between C3 and C7 in patients between 18 and 65 years with neck or radicular pain, neurologic deficits, or signs of cervical myelopathy correlating with the imaging studies in which conservative management failed.

### Exclusion criteria

Exclusion criteria were previous surgery at the index level, systemic or local infection, rheumatoid arthritis, ossified posterior longitudinal ligament, uncontrolled diabetes, and radiological evidence of advanced osteoporosis.

Preoperative functional assessment included the VAS [11] and the NDI [12], which were both patient self-administered. Additionally, the Nurick classification system [13] for myelopathy on the basis of gait abnormalities was applied. Operative time, blood loss, and intra- and postoperative complications were recorded. Dysphagia was evaluated according to Bazaz et al. [4].

Clinical and radiological follow-up was done at one, three, six, and 12 months and the VAS, NDI, Nurick’s myelopathy and Bazaz dysphagia classifications were appraised.

Twenty five patients with 30 pathological disc levels recruited from the outpatient clinic met our inclusion criteria, and were enrolled in this study and no patient was lost during follow-up.

Fusion was primarily assessed by standardized biplanar cervical X-rays and bridging trabecular bone between the endplates and absence of a radiolucent gap between the endplates and graft constituted evidence for osseous union. If in doubt, flexion and extension cervical views should show <1 mm of motion between spinous processes to confirm fusion. If still in doubt, a CT was done not before nine months postoperatively. The preoperative fused segment lordotic angle (FSA) and fused segment height (FSH) were compared to the values at six months and 12 months.

### Surgical technique

All patients received general anesthesia and ceftriaxone 2 g I.V 30 min before incision. The patients were positioned supine on a radiolucent operation table with mild neck extension achieved by placing wrapped towels underneath the interscapular area and placing the occiput in an adequately sized gel ring. The Smith-Robinson anterior cervical approach was used, and the target level was confirmed by a lateral view radiograph. After Cloward retractor and Caspar distraction pin application, routine microscopic discectomy and decompression was done. All patients were operated exclusively by the senior author and they received the Peek Prevail™ cervical interbody device with Zephir™ anterior cervical screws with nitinol anti backout wires (Medtronic Corporation, Memphis, TN, USA). Its superior and inferior screw angle is fixed at 25° ± 3°.

Anterior vertebral body preparation was done with a high-speed diamond burr to carefully match the inferior lip of the superior vertebral body and the superior lip of the inferior vertebral body to the flanges found on both the trial and the implant. The endplates were rasped and then appropriately trialed before inserting the correctly fitting Peek Prevail™ cage into the disc space. Biplanar image intensifier control was done and the starting holes for the locked screws were carefully established with an awl to predetermine the screw trajectory. As the screw is inserted, the nitinol locking wire would deflect and allow the screw to continue until fully inserted. When the screw head is seated, the nitinol locking wire retracts over the screw head preventing its backing out.

### Statistical analysis

Statistical analysis was done with IBM SPSS^TM^ for Windows, Version 18.0. Armonk, NY, IBM Corp. In addition to the standard descriptive statistical analysis, the chi-square for categorical and Student’s *t*-test for nominal data were employed. Throughout the study, the significance level and the confidence interval were set to *p* = 0.05 and CI = 95%, respectively.

## Results

The mean age of the 25 patients involved was 48.96 ± 13.13 years (mean ± *SD*) with 44% of patients (11) in the 40–60 years age group. The gender distribution was 21 (84%)/4 (16%) male/female. There were 20 (80%) single-level and five (20%) double-level affections with disc C5/6 and disc C6/7 involved ten times (33.33%), respectively. C3/4 was affected in six patients (20%) and C4/5 in four patients (13.33%). Mean operative time was 110 min (±42), and average blood loss was 89 cc (±45). The most frequent interbody cage size implanted was 6 mm and the most commonly used screws were 15 mm in length. The neuropathology was located at the cord and the root (radiculomyelopathy) in 11 (44%) patients and purely at the root level in 14 (56%) patients (Table 1).

**Table 1:** Disc-level and neuropathological affection.

Variable	Parameter	
Patients (*n* = 25)	Single	20 (80%)
	Double	5 (5%)
Affected disc levels (*n* = 30)	C3–C4	6 (20%)
	C4–C5	4 (13.33%)
	C5–C6	10 (33.33%)
	C6–C7	10 (33.33%)
Neuropathological affection	Cord and root	11 (44%)
	Root	14 (56%)

The mean preoperative illness duration was 10.32 ± 13.56 months and isolated root affection was present in 14 (56%) of patients with seven (28%) patients being affected on the right and seven (28%) on the left side. Radiculomyelopathic symptoms were found in 11 (28%) patients with radiculation occurring bilaterally in seven (28%) patients and in four (16%) patients occurring on the left side. It was noted that the average duration of preoperative symptoms was longer in the older age groups and in females, which is reflected by the abnormal distribution (*p* < 0.01) and standard deviation (±13.56 months) caused by two females who suffered from preoperative cervical symptoms for 60 months and 36 months, respectively. Earlier studies verified that a short duration of symptoms is a predictor for good surgical outcome [14].

Radiological assessment of fusion was carried out at six months and one year.

**Figure 1:**
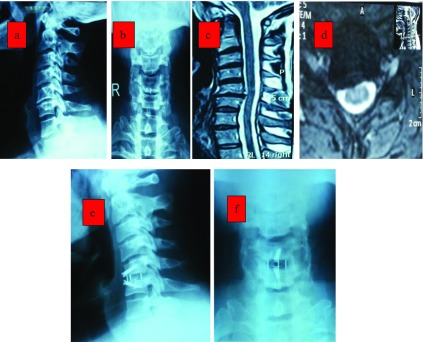
40 years old male with neck pain and right-sided radiculopathy of six months duration. X-rays (a, b) and MRI (c, d) revealed C5–6 prolapsed disc abutting the right exiting C6 nerve root. Follow-up X-ray at 12 months (e, f) showing fusion and maintained disc height.

Successful fusion was achieved in 95% at six months (19 patients) and 100% at one year among the single-level fusion group, 80% at six months (four patients) and 100% at one year among the double-level fusion group (Figure 1).

Lordosis was determined at the operative level by measuring the angle between the superior endplate of the superior vertebral body and the inferior endplate of the inferior vertebral body. The mean FSA was 5.86 ± 1.6 before surgery, 6.8 ± 3.42 at six months after surgery, and 6.1 ± 3.20 at the one-year follow-up examination. There was a significant increase in terms of FSA between preoperative and postoperative at six months and at the 12-month follow-up (*p* < 0.001).

Disc height was measured on preoperative radiographs and determined by measuring the distance from the posterior inferior aspect of the superior vertebral body to the posterior superior corner of the inferior vertebral body. This location was used versus the anterior disc height, as it was felt this would give a better measurement of the increase in vertical neural foramen height and therefore reflect indirectly foraminal decompression.

**Figure 2:**
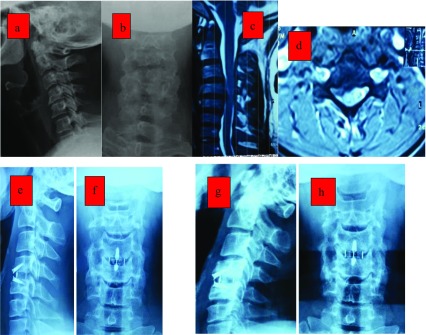
44 years old male with neck pain bilateral radiculopathy and signs of myelopathy. X-rays (a, b). The MRI T2W (c, d) revealed a large C4–5 disc abutting on both sides more to the right side with myelopathic cord changes. Postoperative (e, f) and follow-up X-ray at 12 months (g, h) showing fusion and maintained lordosis.

The mean FSH was 3.2 ± 0.56 mm before surgery, 7.52 ± 1.12 mm at six months after surgery, and 7.38 ± 1.01 mm at one-year follow-up examination. There was a significant increase in terms of the FSH between preoperative and postoperative measurements at six months and at one-year follow-up (*p* < 0.001). No evidence of cage subsidence was noted radiographically (Figure 2).

Comparing the pre-operative and postoperative pain scores at 12 months of the VAS for the neck and upper limb revealed a statistically significant improvement (*p* < 0.001).

Similarly, there was statistical significance regarding the preoperative NDI (*p* < 0.001) and at 12 months postoperatively.

The Nurick Score showed also a statistically significant improvement (*p* < 0.002), when comparing pre-operative and postoperative scores at 12 months (Table 2).

**Table 2:** VAS, NDI and Nurick scores.

Score	Pre-operative	12 months post-op	*p*-value
VAS neck	5.28 + 2.42	0.4 + 0.57	<0.001
VAS upper limb	7.56 + 1.32	0.24 + 0.43	<0.001
NDI	21.64 + 6.76	4.4 + 4.48	<0.001
Nurick score (cord compression) *n* = 11	2.72 + 0.6	1.63 + 0.6	<0.002

VAS, Visual analog scale; NDI, Neck Disability Index.

### Complications

One patient with double-level fusion presented a few days post-operatively with a superficial *Staph. aureus* wound infection that resolved after six days of treatment with I.V antibiotics.

One patient with single-level fusion exhibited delayed fusion at nine months and another patient with double-level fusion showed delayed fusion at 12 months. No revision surgery or posterior cervical instrumentation was needed as both patients united without consequences after three further months.

Five patients reported dysphagia immediately postoperatively and were classified according to Bazaz score. Dysphagia-related symptoms were graded depending on the patient’s state as none (no episodes of swallowing problems), mild (rare episodes of dysphagia), moderate (occasional swallowing difficulty with specific food), and severe (frequent difficult swallowing with majority of food).

Three patients with mild transient dysphagia showed complete improvement at two weeks, and two cases of moderate dysphagia resolved at five weeks the latter being two-level ACDFs. None of these five patients complained about long-term dysphagia.

Three patients reported of upper limb tingling and numbness, but they improved after three months of conservative treatment with NSAID and physiotherapy (Table 3).

**Table 3:** Complications.

Complications	*n*	% of cases
Local infection	1	4
Tingling and numbness	3	12
Dysphagia	5	20
Delayed fusion	2	8
Lost to follow-up	1	4

## Discussion

ACDF is the operative procedure of choice for degenerative disc disease and cervical spondylosis associated with radiculopathy or myelopathy, hitherto the ideal implant from the biological and biomechanical points of view has yet not been determined and it depends largely on the surgeon’s preference and training [15,16].

The use of a stand-alone cage has been questioned by many authors for its efficacy and safety. Loss of lordosis and a high rate of cage subsidence have been reported frequently [15]. On the other hand, dysphagia, stress-shielding, and subsequent adjacent-level affection manifested as adjacent-level ossification have been reported in plate augmented ACDF procedures despite the improved plate designs [17,18].

The hypothetical enhanced stability provided by cervical plates is crucial for improving fusion rates, maintaining sagittal alignment and long-term functional outcome [19].

Nevertheless, there has been no substantial evidence for the superiority of constrained or dynamic cervical plate systems over stand-alone cages regarding radiological and functional outcome measures, which is reflected by the wide diversity of implants used in ACDF [15,18].

Majid et al. compared the biomechanical performance in a cadaveric model of an anatomically profiled two-screw integrated plate-spacer, four-screw integrated plate-spacer, a traditional cervical spacer only, and a cervical spacer and anterior cervical plate construct. No significant differences were found in motion between any of the instrumented conditions in any of the loading conditions [20].

Stein et al. compared the integrated screw and cage system against locked anterior plate fixation at C5–C6 in human cadaveric spines and reported that the integrated screw and cage implant provided almost similar biomechanical stability compared to traditional plating [21].

In 2011, Scholz et al. reported of 34 patients with single- or multi-level ADCF in whom the ZERO-P^®^ Spacer (DePuy Synthes, Raynham, MA, USA) was used. They achieved good fusion rates and only one patient (2.9%) showed chronic dysphagia [22].

In 2013, Yang et al. analyzed the radiological and clinical outcomes of multilevel anterior cervical discectomy and fusion with the anchored spacer and anterior plate fixation. No statistically significant difference existed in the fusion rate at six-month follow-up, and all patients achieved solid osseous fusion and had a good clinical outcome. A significant improvement was observed in lordosis in both groups, which was maintained well at the final follow-up. No cage subsidence or fusion segmental kyphosis was observed. This improvement was attributed to disc height restoration. The dysphagia rate was lower, the Swallowing Quality-of-Life condition was better, and the mean thickness of the prevertebral soft tissue was thinner in the anchored spacer group [18].

Hofstetter et al. compared the incidence of dysphagia in 70 patients treated with ACDF with an anterior plate (*n* = 35) versus an anchored cage (*n* = 35). Increased prevertebral swelling in the anterior plate group verified radiologically remained up to six months. There were also 7 (20%) patients in the anterior plate group that complained of dysphagia in contrast to only one (2.85%) in the zero profile group [23].

In the underlying study, radiological assessment of fusion was carried out at six months and one year. Successful fusion was achieved in 95% at six months (19 patients) and 100% at one year among the single-level fusion group, 80% at six months (four patients) and 100% at one year among the double-level fusion group. No evidence of screw or implant migration was observed. No evidence of adjacent-level degeneration was noted in any of our patients.

In 2013, Barbagallo and colleagues reported on 32 patients treated with an anchored zero-profile stand-alone cage with so far the longest follow-up of four years. NDI showed a statistically significant improvement (*p* < 0.01) and mean arm pain VAS score decreased from 79 to 41. X-rays and CT demonstrated, respectively, a 94.5% and a 92% fusion rate. Three patients complained of moderate and two of mild transient dysphagia (15.5%). No device-related complications occurred, and no fractures were reported. They found the zero profile device to be safe and effective, even on multilevel cases [24].

In 2014, Scholz et al. [25] reported on the safety and efficacy using a new zero-profile stand-alone cage with integrated angle-stable fixation in single- and multilevel anterior cervical fusions based on 53 consecutive patients and 97 levels operated. The fusion rate reached 97% and three out of 45 patients (6.6%) complained of mild dysphagia at 24 months follow-up (Table 4).

**Table 4:** Comparison between different studies.

Study	Year	No. of cases	No. of levels	Follow-up	Fusion rate	Dysphagia
Yang et al. [18]	2012	23	23	1 year	91% at 6 months, 100% at 1 year	17%
Hofstetter *et al.* [23]	2015	35	35	6 months	94%	3%
Barbagallo et al. [24]	2013	32	32	4 years	92%	15%
Scholz et al. [25]	2014	50	87	1 year	97%	10%
Current study	2017	25	30	1 year	92% at 6 months, 100% at 1 year	20%

The underlying study of 25 patients shows a significant statistical difference in the NDI and VAS for neck and upper limb pain comparing pre-operative and post-operative 12-month scores (*p* < 0.001). Similarly, there is a significant statistical difference (*p* < 0.002) in the Nurick Score on comparing pre-operative and post-operative scores. Our results were similar regarding fusion rates, cervical alignment, functional outcome, operative time, and blood loss compared to the above-mentioned studies.

Similarly, in this study five patients (20%) reported dysphagia postoperatively, three cases (12%) of mild transient dysphagia resolved in two weeks, and two cases (8%) of moderate dysphagia resolved in five weeks both were two-level ACDFs. None of these five patients complained about long-term dysphagia.

Confounding factors of this prospective study are the small number of patients, mono-centric, single-surgeon nature, and the lack of a comparative stand-alone cage group. Despite the use of standardized radiological protocols, linear distance measurement errors due to parallax and patient mal-positioning may affect the FSA and FSH measurements. Furthermore, adjacent-segment degenerations are usually not detectable in a follow-up period of 12 months. This necessitates larger multi-centric studies with longer follow-up to reach an evidence-based consensus regarding zero profile implants.

## Conclusion

A zero-profile device with integrated locking screw fixation provides biomechanical stability and fusion rates with excellent outcomes for one- and two-level ACDFs. Advantages include low rates of dysphagia, decreased operative time, restoration of cervical lordosis and disc height, and lack of cage subsidence or screw back out. Larger and longer multi-centric studies are needed to detect adjacent-level degeneration and compare it to other established devices.

## Declarations section

### Availability of data and materials

The datasets used and/or analyzed during the current study are available from the corresponding author on reasonable request.

## References

[1] Song K-J, Taghavi CE, Hsu MS, Lee K-B, Kim G-H, Song J-H (2010) Plate augmentation in anterior cervical discectomy and fusion with cage for degenerative cervical spinal disorders. Eur Spine J 19, 1677. 20376680 10.1007/s00586-010-1283-3 PMC2989224

[2] Korinth M (2008) Treatment of cervical degenerative disc disease – Current status and trends. Zentralbl Neurochir – Cent Eur Neurosurg 69, 113–124. 10.1055/s-2008-1081201 18666050

[3] Kaiser MG, Haid RW, Subach BR, Barnes B, Rodts GE (2002) Anterior cervical plating enhances arthrodesis after discectomy and fusion with cortical allograft. Neurosurgery 50, 229–236. 11844257 10.1097/00006123-200202000-00001

[4] Bazaz R, Lee MJ, Yoo JU (2002) Incidence of dysphagia after anterior cervical spine surgery: a prospective study. Spine (Phila Pa 1976) 27, 2453–2458. 12435974 10.1097/00007632-200211150-00007

[5] Riley LH, Skolasky RL, Albert TJ, Vaccaro AR, Heller JG (2005) Dysphagia after anterior cervical decompression and fusion: Prevalence and risk factors from a longitudinal cohort study. Spine (Phila Pa 1976) 30, 2564–2569. 16284596 10.1097/01.brs.0000186317.86379.02

[6] Fountas KN, Kapsalaki EZ, Nikolakakos LG, Smisson HF, Johnston KW, Grigorian AA, Lee GP, Robinson JS (2007) Anterior cervical discectomy and fusion associated complications. Spine (Phila Pa 1976) 32, 2310–2317. 17906571 10.1097/BRS.0b013e318154c57e

[7] Park JB, Cho YS, Riew KD (2005) Development of adjacent-level ossification in patients with an anterior cervical plate. J Bone Jt Surg – Ser A 87, 558–563. 10.2106/JBJS.C.01555 15741622

[8] Yang JY, Song HS, Lee M, Bohlman HH, Riew KD (2009) Adjacent level ossification development after anterior cervical fusion without plate fixation. Spine (Phila Pa 1976) 34, 30–33. 19127159 10.1097/BRS.0b013e318190d833

[9] Scholz M, Reyes PM, Schleicher P, Sawa AGU, Baek S, Kandziora F, Marciano FF, Crawford NR (2009) A new stand-alone cervical anterior interbody fusion device: Biomechanical comparison with established anterior cervical fixation devices. Spine (Phila Pa 1976) 34, 156–160. 19139665 10.1097/BRS.0b013e31818ff9c4

[10] Zhou J, Li X, Dong J, Zhou X, Fang T, Lin H, Ma Y (2011) Three-level anterior cervical discectomy and fusion with self-locking stand-alone polyetheretherketone cages. J Clin Neurosci 18, 1505–1509. 21924914 10.1016/j.jocn.2011.02.045

[11] Huskisson EC (1982) Measurement of pain. J Rheumatol 9, 768–769. 6184474

[12] Vernon H, Mior S (1991) The Neck Disability Index: a study of reliability and validity. J Manipulative Physiol Ther 14, 409–415. 1834753

[13] Nurick S (1972) The pathogenesis of the spinal cord disorder associated with cervical spondylosis. Brain 95, 87–100. 5023093 10.1093/brain/95.1.87

[14] Peolsson A, Peolsson M (2008) Predictive factors for long-term outcome of anterior cervical decompression and fusion: A multivariate data analysis. Eur Spine J 17, 406–414. 18084782 10.1007/s00586-007-0560-2 PMC2270379

[15] Song KJ, Taghavi CE, Lee KB, Song JH, Eun JP (2009) The efficacy of plate construct augmentation versus cage alone in anterior cervical fusion. Spine (Phila Pa 1976) 34, 2886–2892. 19949367 10.1097/BRS.0b013e3181b64f2c

[16] Pitzen TR, Chrobok J, Štulik J, Ruffing S, Drumm J, Sova L, Kučera R, Vyskočil T, Steudel WI (2009) Implant complications, fusion, loss of lordosis, and outcome after anterior cervical plating with dynamic or rigid plates: Two-year results of a multi-centric, randomized, controlled study. Spine (Phila Pa 1976) 34, 641–646. 19287352 10.1097/BRS.0b013e318198ce10

[17] Park JB, Cho YS, Riew KD (2005) Development of adjacent-level ossification in patients with an anterior cervical plate. J Bone Jt Surg - Ser A 87, 558–563. 10.2106/JBJS.C.01555 15741622

[18] Yang L, Gu Y, Liang L, Gao R, Shi S, Shi J, Yuan W (2012) Stand-alone anchored spacer versus anterior plate for multilevel anterior cervical diskectomy and fusion. Orthopedics, 35, e1503–e1510. 23027488 10.3928/01477447-20120919-20

[19] Saphier PS, Arginteanu MS, Moore FM, Steinberger AA, Camins MB (2007) Stress-shielding compared with load-sharing anterior cervical plate fixation: a clinical and radiographic prospective analysis of 50 patients. J Neurosurg Spine 6, 391–397. 17542503 10.3171/spi.2007.6.5.391

[20] Majid K, Chinthakunta S, Muzumdar A, Khalil S (2012) A comparative biomechanical study of a novel integrated plate spacer for stabilization of cervical spine: An in vitro human cadaveric model. Clin Biomech 27, 532–536. 10.1016/j.clinbiomech.2011.12.013 22244511

[21] Stein MI, Nayak AN, Gaskins RB, Cabezas AF, Santoni BG, Castellvi AE (2014) Biomechanics of an integrated interbody device versus ACDF anterior locking plate in a single-level cervical spine fusion construct. Spine J 14, 128–136. 24231054 10.1016/j.spinee.2013.06.088

[22] Scholz M, Schnake KJ, Pingel A, Hoffmann R, Kandziora F (2011) A new zero-profile implant for stand-alone anterior cervical interbody fusion. Clin Orthop Relat Res 469(3), 666–673. 20882376 10.1007/s11999-010-1597-9 PMC3032850

[23] Hofstetter CP, Kesavabhotla K, Boockvar JA (2015) Zero-profile anchored spacer reduces rate of dysphagia compared with ACDF with anterior plating. J Spinal Disord Tech 28, E284–E290. 23429316 10.1097/BSD.0b013e31828873ed

[24] Barbagallo GMV, Romano D, Certo F, Milone P, Albanese V (2013) Zero-P: A new zero-profile cage-plate device for single and multilevel ACDF. A single Institution series with four years maximum follow-up and review of the literature on zero-profile devices. Eur Spine J 22, S868–S878. 24061968 10.1007/s00586-013-3005-0 PMC3830046

[25] Scholz M, Schelfaut S, Pingel A, Schleicher P, Kandziora F (2014) A cervical “zero-profile” cage with integrated angle-stable fixation: 24-months results. Acta Orthop Belg 80, 558–566. 26280730

